# Aerobic Capacity in Adults with Congenital Heart Disease: More than VO_2peak_, a Follow-Up Study

**DOI:** 10.3390/life12122118

**Published:** 2022-12-15

**Authors:** Kelly Ferri, Ignasi Gich, Myriam Guerra-Balic, Guillermo R. Oviedo, Maite Doñate, Mireia Parra, Bàrbara Carbonell-Prat, Laura Dos-Subirá, Ricard Serra-Grima

**Affiliations:** 1 FPCEE—Blanquerna, Ramon Llull University, 08022 Barcelona, Spain; 2 CIBER Epidemiology and Public Health (CIBERESP), 08025 Barcelona, Spain; 3 Cardiology Department, Santa Creu i Sant Pau Hospital, IIB Sant Pau, 08025 Barcelona, Spain; 4 Adult Congenital Heart Disease Unit, Vall d’Hebron University Hospital, 08035 Barcelona, Spain

**Keywords:** congenital heart disease, aerobic capacity, follow-up, adults

## Abstract

To control the development of people with congenital heart disease (CHD), it is important to follow their aerobic capacity (AC), especially when they exercise. This research aimed to study the progress of AC during a follow-up of adults with CHD. This is a longitudinal study which involved 127 adults with a mean age of 33.8 (11.1) years (57.5% female; 75 moderate CHD and 52 complex CHD) who had undergone two cardiopulmonary exercise tests (CEPT) in at least one year between the first and the second test. The AC and exercise performance (EP) (duration of exercise time, velocity and percentage of grade) were assessed using a ramp protocol over a treadmill. In a mean of 4.5 (2.0) years of follow-up, there was a significant decrease in AC. The VO_2peak_ at baseline was 27.8 (27.7) mL/kg/min (82.9% (20.3%) predicted) versus 26.6 (7.8) mL/kg/min (79.3% (20.8%) predicted) at the end of follow-up. This decline was independent of the body weight increase. There was no significant difference in HR_peak_ and EP among periods. These results suggest a sign of favorable evolution of adults with CHD. More research is needed to study different factors that could contribute to AC reduction.

## 1. Introduction

Evolution in pediatric diagnostic and surgical techniques has resulted in a significant impact on increasing survival and reducing mortality in children with congenital heart disease (CHD) [1,2]. Newborns with tetralogy of Fallot or transposition of the great arteries have reached adulthood with a good quality of life without relevant incidents [3].

Aging is associated with a decline in aerobic capacity (AC) and exercise performance (EP). This is a physiological phenomenon. Adults with CHD (ACHD) often overestimate their physical capabilities and under-report limitations [4]. The decline in AC may occur imperceptibly over many years and will be influenced by (1) type of CHD, (2) activity lifestyle during adolescence and adulthood and (3) body weight control [2].

Müller and colleagues [5] suggested that the only way to detect a progressive change in AC is serial exercise testing. In this case, the most recognized methods to exercise testing are the six minute walk test (6MWT) and the cardiopulmonary exercise testing (CPET). The 6MWK is a simple and inexpensive test that provides information about AC based on the distance walked and the vital signs during the test. On the other hand, the CPET is a more complex method that provides AC information based on physiological and cardiologic parameters during a maximum exercise test [6]. 

One of the objectives of follow-up using consecutive CPET is to assess how much decline occurs in AC measured by VO_2peak_.

The purpose of this study is to describe our experience with ACHD attending a CPET department and study their evolution of AC [VO_2peak_], EP [duration of exercise time, velocity and percentage of grade], and body weight changes. 

## 2. Materials and Methods

### 2.1. Study Design and Participants

We retrospectively reviewed all CPET studies performed in ACHD population between February 2010 and March 2021 in two institutions. Data were obtained by the ACHD unit of Vall d’Hebron Hospital, and at the Santa Creu i Sant Pau Hospital. The inclusion criteria were patients older than 16 years old with CHD who had two or more consecutive CPET separated by at least 1 year.

Initially, a total of 151 patients were included. However, 24 of them were excluded based on the following exclusion criteria described below.

Six patients were younger than 16 years at first CPET; nine patients had a peacemaker implant that limited heart rate during exercise (eight patients had stimulation mode in DDD and one in VVI); three patients who had residual heart lesions but their initial diagnosis was not a CHD (e.g., rheumatic aortic valve disease); four patients that stopped exercising at the first or the second CPET before reaching their cardiovascular limit, defined as respiratory exchange ratio at peak exercise < 1.15 or heart rate at peak exercise < 85% of the predicted [7,8]; one patient with a severe eating disorder (bulimia) and one patient who had interrupted CPET due to arterial hypertension on effort. 

Finally, a total of 127 patients were included and provided informed consent.

According to AHA/ACC guidelines [2], patients were classified depending on their CHD severity. (1) Moderate (e.g., biventricular repairs including tetralogy of Fallot, atrioventricular canal defects, and significant valvular issues) and (2) Complex (e.g., patients with single-ventricle physiology, transposition of the great arteries, pulmonary atresia with or without ventricular septal defect, as well as cyanotic lesions) [2].

An expert cardiologist performed this classification without direct relationship with the participants, and without knowledge of the CPET results, to avoid expectation and maintain a blind classification. 

The study protocol was approved by the FPCEE—Blanquerna institutional research board (protocol nº1718005D) and follows the Helsinki guidelines for ethical behavior [9].

### 2.2. Instruments and Procedure

#### 2.2.1. Anthropometric Measurements

Height was measured to the nearest 0.1 cm using a stadiometer (Seca 225, Seca, Hamburg, Germany). Weight was measured to the nearest 0.1 kg on a digital scale (Seca 861, Hamburg, Germany) with the subject wearing lightweight clothing and no shoes. Body mass index (BMI) was calculated as weight in kilograms divided by height in squared meters (kg/m^2^) [10]. As recommended by the WHO [11], patients were classified based on BMI as underweight (<18.5 kg/m^2^), normal weight (18.5–24.9 kg/m^2^), overweight (≥25–29.9 kg/m^2^), and obese (≥30 kg/m^2^).

#### 2.2.2. Cardiopulmonary Exercise Testing (CPET)

All tests were conducted during the afternoon at a room temperature of 21–22 °C and relative physical humidity between 55 and 65%. The CPET was performed on a treadmill (Schiller^®^ MTM–1500 MED) using a progressive protocol. The participants started walking at 3 km/h for two minutes, after which the speed increased 0.3 km/h and 1.4% grade every minute to a maximum of 12% until exhaustion of participants. Participants were asked every 2/3 min about new appearance of symptoms (such as dyspnea, angina, general discomfort or nausea), and were verbally encouraged to push themselves [12].

Heart rate was obtained using a 12-lead electrocardiogram (Schiller^®^ Cardiovit CS–200) and blood pressure was measured using a sphygmomanometer (Sphygmomanometer Riester^®^ model) at rest, at the end of each stage, at peak exercise, and during recovery (at the first, third and fifth minute after finishing the exercise).

Physiological parameters of CPET as relative VO_2peak_ (mL/kg/min) and VE/VCO_2_ slope, were obtained breath-by-breath with an automatic gas analysis system (Ganshorn^®^ Power–cube). The automatic system calculated this parameter based on the Fick method for the estimation of cardiac output from the uptake VO_2_ and the arterial mixed venous O_2_ [13].

The expected VO_2peak_ (mL/kg/min) for men and women was obtained with the automatic gas analysis system (Ganshorn^®^ Power–cube). The system calculated this parameter based on the equation published by Wassermann et al. [13], considering various influencing factors, including weight (measured and expected), mode of exercise (treadmill) and sex. We compared the VO_2peak_ with the expected VO_2peak_ through a percentage of the difference between the observed and the expected.

#### 2.2.3. Echocardiographic Doppler

For each patient, we collected data from the echocardiographic study closest to the date of the CPET period 1 and period 2. This data was collected offline from EchoPAC V.201, GE Healthcare. 

Echocardiography included left or systemic ventricular ejection fraction (EF). The ventricular ejection fraction, in single ventricle physiology (patients with Fontan), was collected by magnetic resonance imaging, according to cardiological recommendations.

#### 2.2.4. International Physical Activity Questionnaire (IPAQ)

The IPAQ was applied to 183 participants; 39 were in a subgroup of patients for whom we obtained prior CPET data. In this subgroup of 39 participants, we assessed their physical activity (PA) at follow-up. The IPAQ short form [14] was used to assess PA levels within the last seven days. The questionnaire can obtain the objective measure equivalent to the energy spent in PA (metabolic equivalent task MET-min/week) reported. The IPAQ includes three questions related to vigorous, moderate and light activity. According to the WHO, acceptable values for adults are above 600 MET-min/week [15]. The IPAQ shows acceptable reliability and validity and it is a valid measurement tool for assessing PA levels in individuals with CHD [16].

#### 2.2.5. Statistical Analysis

Descriptive statistics were calculated for all variables. Continuous variables were expressed as mean and standard deviation (SD) or median (interquartile range) when variables were not normally distributed. Categorical variables were described as frequency and percentage. Normal distribution of variables as change in VO_2peak_, change in body weight and change in EF, was tested by a Kolmogorov–Smirnov test, in addition to visual inspection.

Two-way analysis of variance (ANOVA) was used to compare continuous variables between baseline and follow-up in moderate and complex CHD severity, adjusting for sex (men/women) and age of the participants.

Associations of changes in HR_peak_ and follow-up medication; PA (only at follow-up) and VO_2peak_ were assessed using Pearson correlation.

Statistical analysis was performed with SPSS version 26 for Windows (IBM SPSS Inc., Armonk, New York, NY, USA). Differences were considered statistically significant if *p*-values ≤ 0.05.

## 3. Results

Overall, 127 participants were included in the current analysis. The severity of congenital heart disease distribution was 59.0% moderate and 41.0% complex (Table 1). The most common diagnosis was transposition of the great arteries, repaired with an atrial switch procedure (seening or mustard repairs) (38.1%) followed by tetralogy of Fallot (31.8%) and pulmonary valve stenosis (19.1%). The complete description of all diagnostic groups is available in Table 2.

The mean follow-up duration between baseline and final CPET was 4.5 (2.0) years. The median age of the study group was 33.8 years and 57.5% of participants were female (Table 3). At baseline, participants with moderate CHD were significantly older than participants with complex CHD (35.7 [IQR 33.0–38.4] vs. 31.0 [IQR 28.4 × 33.7] years; *p* = 0.02). At baseline, 50% of participants were in the New York Heart Association (NYHA) functional class I and 50% were in class II. At follow-up, 33.3% of participants were in class I and 84.6% in NYHA class II. 

At baseline, eight participants had AF/flutter; two of them went through ablation before the first CPET. At follow-up, nine participants had AF/flutter; four of them were the same participants with AF/flutter at baseline (none had ablation recurring). Regarding the new cases of AF/flutter at follow-up, one out of five participants went through ablation between the first and second CPET. All participants had controlled AF/flutter with no impact on the physiological parameters of CPET.

### 3.1. Longitudinal Changes in the Different Groups of CHD

#### 3.1.1. The VO_2peak_

There was a significant decline in the VO_2peak_ from 27.8 (7.7) mL/kg/min to 26.6 (7.8) mL/kg/min at follow-up. This decrease converted to percent VO_2_ predicted was from 82.9% (20.3%) to 79.3% (20.8%) at follow-up. 

The decline in VO_2peak_ is statistically significant in both severity CHD groups during follow-up; *p* = 0.003 (Table 3). The moderate group had a lower decline than the complex group (−0.5 (5.6) mL/kg/min vs. −2.3 (4.7) mL/kg/min, respectively).

In order to understand the evolution of VO_2peak_, we investigated how the VO_2peak_ was changed during follow-up. A total of 56.5% of participants had a decreased VO_2peak_, 33.9% of participants increased and 9.6% of participants maintained the VO_2peak_ at follow-up. 

The different ranges of decreased VO_2peak_ were: (a) between 1 and 4 mL/kg/min: 33.0% of participants; (b) between 5 and 10 mL/kg/min: 18.8% of participants and (c) >10 mL/kg/min: 4.7% of participants. The different ranges of increased VO_2peak_ were (a) between 1 and 4 mL/kg/min: 21.3% of participants and (b) >5 mL/kg/min: 12.6% of participants. 

Among the participants who increased more than 5 mL/kg/min of VO_2peak_, seven of them began to practice PA; seven had a replaced pulmonary valve intervention and the remaining two had an aortic valve replacement intervention between two CPETs. 

There were no significant differences in EP (time exercise, percentage slope and maximal velocity) between baseline and follow-up or between severities CHD groups (Table 3). Mean exercise time was 12.8 min, the mean percentage slope was 11.4% and the mean maximal velocity was 6.2 km/h at baseline. 

**Figure 1:**
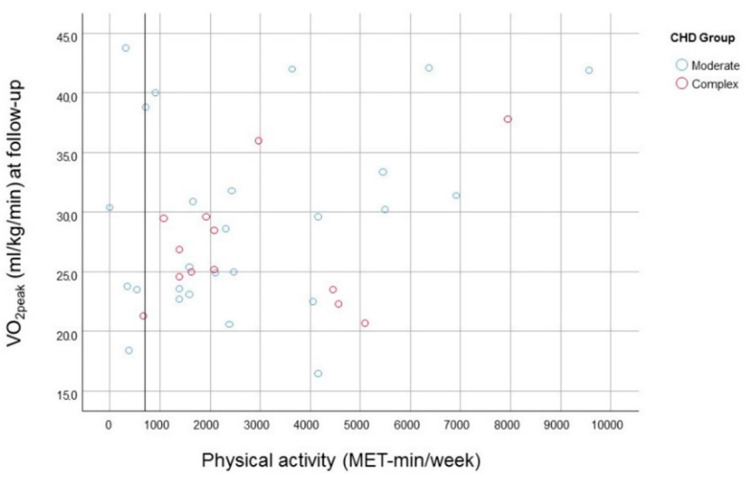
Aerobic capacity (VO_2peak_) depending on physical activity (MET-min/week) assessed by IPAQ in the follow-up.

In a subgroup of 39 participants with measures of PA by IPAQ, the median PA reports as assessed by IPAQ at follow-up were 2079.0 (IQR 2772.0) MET-min/week, oscillating between 0 and 9564.0 MET-min/week (Table 3). From 39 participants that answered the IPAQ questionnaire, 13 (33.3%) reported health-enhancing PA, 19 (48.7%) reported minimally active, and 7 (17.9%) reported inactivity. However, the PA data have not been sufficient to study the evolution in the statistical analysis model. The Pearson correlates showed a positive trend between PA (MET-min/week) and AC at the follow-up assessment (*p* = 0.06) (Figure 1).

#### 3.1.2. Maximum Heart Rate (HR_peak_) in Exercise

Mean HR_peak_ was 160.3 beats per minute (bpm) at baseline and 158.8 bpm at follow-up. There is a significant difference in HR_peak_ between CHD groups (*p* = 0.04). The moderate CHD group achieved an HR_peak_ higher than the complex CHD group in both periods (Table 3). However, no significant differences were found between periods.

**Figure 2:**
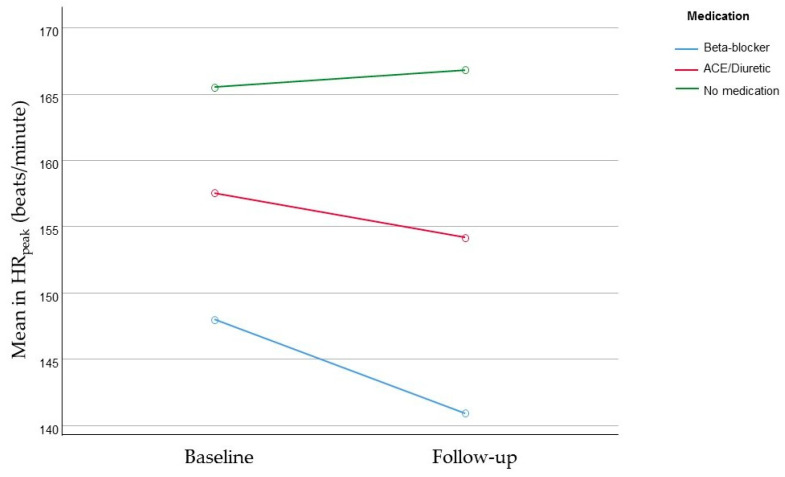
Evolution in HR_peak_ according to the use of medication.

The reduction in the HR_peak_ in both CHD groups has been associated with the increased use of beta-blocker and diuretic medication during the follow-up; *p* = 0.04 (Figure 2). At baseline, 16 (12.5%) participants used a beta—Bisoprolol (n = 4), Atenolol (n = 3), Carvedilol (n = 3), others that combined a beta-blocker with an antiarrhythmic (n = 6); and 10 (7.8%) participants used an Angiotensin-converting enzyme (ACE)/diuretics—Enalapril (n = 9), others (n = 2). During follow-up, there was an increase resulting in 32 (25.1%) participants taking beta-blocker—Bisoprolol (n = 13), Carvedilol (n = 5), Atenolol (n = 4), Amiodarone (n = 3), or others that combined a beta-blocker with an antiarrhythmic (n = 7); and 19 (14.9%) participants used ACE/diuretics—Enalapril (n = 9), Losartan (n = 3), others (n = 7). 

#### 3.1.3. VE/VCO_2_ Slope

The mean of the VE/VCO_2_ slope was 26.9 (6.6) at baseline and 28.5 (6.7) at follow-up. There was a significant increase in VE/VCO_2_ slope for both CHD groups. The mean of the increase was 1.57 (4.9) at follow-up. 

The complex CHD group had a significantly higher mean VE/VCO_2_ than the moderate CHD group in both periods (Table 3). 

There were five participants with a VE/VCO_2_ higher than 40 (range 42.1 to 62.7) at baseline. Among them, two participants decreased VE/VCO_2_ to normal values at follow-up and three participants with a univentricular heart (VO_2peak_ between 12.6 and 18.9 mL/kg/min and %V0_2_ predicted below 40%) were referred for cardiac transplant during follow-up.

#### 3.1.4. Ejection Fraction (EF)

The percentage of participants with reduced EF (<50%) was lower in the moderate group than the complex group in both periods (2.8% of participants vs. 53.3%, respectively at baseline; and 9.3% of participants vs. 55.6% at follow-up). The mean percentage of EF in the moderate CHD group was significantly higher than in the complex CHD group in both periods (64% vs. 50%, respectively, at baseline and 60% vs. 48% at follow-up; *p* < 0.001). 

There was a significant decrease in EF in both severity CHD groups during follow-up. The mean decrease in EF at follow-up was significantly higher in the moderate CHD group than the complex CHD group (−3.7% (7.7) vs. −3.0% (9.7), respectively); *p* < 0.001 (Table 3). A total of 60.8% of participants decreased their EF at follow-up, 33.7% of participants increased and 5.5% maintained the same percentage of EF.

When we compared EF by severity, we observed that a higher baseline EF was associated with a greater decrease in EF during the follow-up period. However, the reduction in EF has not been associated with the decrease in AC (*p* = 0.96).

### 3.2. Longitudinal Association between Body WEIGHT Changes and Aerobic Capacity

At baseline, the majority of participants had normal body weight (59.4%), while 29.9% of participants were overweight, 7.8% were underweight and 2.3% were classified as obese (Table 3). 

The mean amount of body weight change during follow-up was an increase of 1.5 (5.3) kg., a total of 58.3% of participants increased their body weight, 25.2% decreased and 16.5% maintained their body weight during follow-up. 

The two-way ANOVA showed that the body weight increase is significant during follow-up (*p* = 0.001) and it is independent of the severity (*p* = 0.41). 

**Figure 3:**
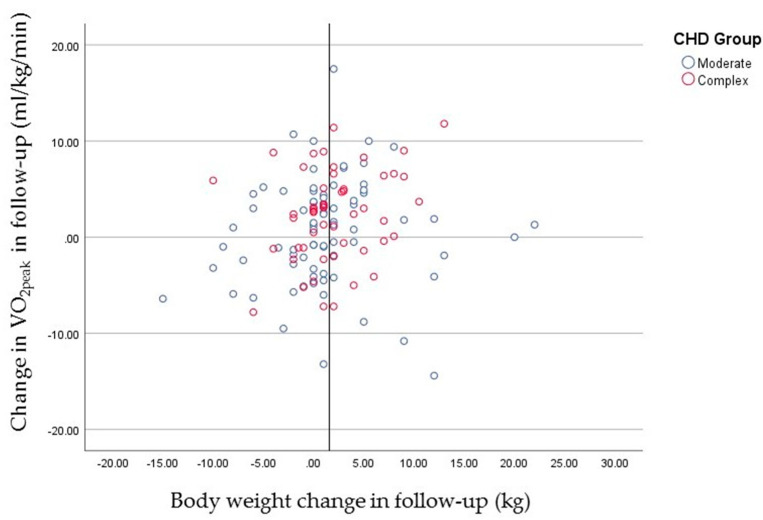
Aerobic Capacity (VO_2peak_) change depending on body weight change.

In Figure 3, we observed the changes in body weight and their relation with the VO_2peak_ at follow-up. The most common change was an increase between 1 and 5 kg at follow-up (42.2% of the participants) followed by the maintenance on body weight (16.5% of the participants) and a decrease between 1 and 5 kg (16.5% of participants). 

Pearson’s correlation revealed the small negative association between body weight and increased AC (measured by VO_2peak_) in both CHD groups, regardless of gender or age. Despite this correlation, in general there was only a trend (*p* = 0.15). The tendency is more evident in the complex CHD group (*p* = 0.07) than the moderate CHD group (*p* = 0.57). 

## 4. Discussion

CPET is a method to assess AC permitting physicians to take objective medical decisions and give specific recommendations about physical exercise. 

This observational study assessing two different CPET results from a sample of 127 CHD patients has given us a wide range of information about changes in AC during follow-up in this population.

The aim of this study was to describe our experience with ACHD attending a CPET department and study their AC evolution (VO_2peak_), EP (duration of exercise time, velocity and percentage of grade), and assess if the body weight changes could worsen the AC of adults with moderate and complex CHD. 

The current study showed a reduction in AC in moderate and complex severity groups during the follow-up. These findings are in accordance with the longitudinal study of Müller, et al. [5]. These authors described a progressive but slow decline in VO_2peak_ −1.75 (4.42) mL/kg/min in 2.5 years. In our study, this decline was −1.27 (5.33) mL/kg/min in 4.5 years. The slow reduction in VO_2peak_ observed in our cohort could be interpreted as clinical stabilization in the functional impact of heart disease due to the short follow-up time.

Aerobic capacity is correlated with exercise capacity. In this context, the VO_2peak_ obtained by CPET is the most objective clinical parameter of AC assessment and estimating exercise capacity. A patient is considered to reach their VO_2peak_ when they reach exhaustion and cannot continue with physical exercise. The physiological mechanism to reach exhaustion is double; it depends on the central factor (e.g., heart rate) and the peripheral factor (characteristics of the skeletal muscle: body weight and exercise adaptations).

Based on the physiological mechanism, there are some arguments that could explain the slight reduction in VO_2peak_. One of them is related to a central factor and it refers to the maintenance of the HR_peak_ reached during exercise. Because of a reduced ejection fraction that some CHD patients have, treatment with beta-blockers is relatively frequent. In fact, we observed a greater number of patients taking beta-blockers at second CPET. This therapeutic measure could favorably contribute to the VO_2peak_ response at follow-up.

Moving the peripheral factor, an important variable that influences cardiac output is body weight. Absolute VO_2_ (L/min) reduction at follow-up was not significant in our sample. However, for VO_2peak_, the difference between periods is relevant. This occurs because body weight is the denominator in the mathematical equation for obtaining relative VO_2peak_ [VO_2_ (L/min)/body weight (kg)] [13]. For example, a patient weighing 60 kg with an initial absolute VO_2_ consumption of 1800 L/min in the first test will have a consumption of VO_2peak_ relative 30 mL/kg/min (1800/60). If this patient in the second test reaches the same absolute consumption of 1800 L/min but has increased his body weight to 65 kg, his VO_2peak_ relative consumption will be lower, reaching 27 mL/kg/min (1800/65). In this way, VO_2peak_ consumption will be lower even without a deterioration in cardiac function. This mathematical explanation of the equation is evident in the inverse correlation that we found between body weight and VO_2peak_ in our cohort. Nevertheless, the reduction in VO_2peak_ at follow-up observed in our cohort was independent of body weight changes. We believe that this result could be explained by the fact that the absolute weight gain was not very high (range between 1–5 kg in 4.5 years).

Brida et al. [17], in a study with more than 3000 ACHD patients, suggested that temporary changes in body weight could provide prognostic information, especially when associated with decreased physical capacity. The authors also concluded that symptomatic ACHD patients (NYHA >= 2) may fare better when maintaining a higher BMI (between 24–30 kg/m^2^). We believe that even with absolute weight gain, the maintenance of a healthy BMI range may have contributed to the low VO_2peak_ reduction in our cohort.

Willinger et al. [18] published a review including 6657 ACHD patients and they observed an overweight prevalence in a range from 22 to 53%, while obesity was reported in a range between 7% and 26%. In our cohort, only 29.9% were overweight and 2.3% were obese in the baseline. The changes in body weight have been significant during follow-up. However, the average BMI has remained within normal values, keeping the majority of the sample (60.6% of participants) with normal weight during follow-up. 

Some studies have described that patients with CHD are more likely to be involved in their health care and control of cardiovascular risk factors [19,20]. In this way, we highlight the importance of health education and the promotion of an active lifestyle, with the aim that each patient can understand how the peripheral factor can affect their physical capacity even when the body weight gain is only between 1 and 5 kg.

We also assessed the relationship between EF and AC. In our sample, the decline in EF has been shown to be significant during follow-up. When we compared EF by CHD severity, we observed that a higher baseline EF was associated with a greater decrease in EF during the follow-up period. However, the reduction in EF has not been associated with the decrease in AC. This finding is in accordance with Cuypers J. et al. [21]. The authors presented results of EF and AC in a long-term follow-up study with a cohort of TGA participants evaluated systematically every 10 years starting from birth, for nearly 40 years. The study showed a progressive decrease in EF in the last 10 years of follow-up (between 30-40 years old); however, AC remained stable. 

The VE/VCO_2_ slope is another CPET systematically evaluated in our cohort of patients. It describes the relationship between ventilation and CO_2_ production, indicating the state of ventilatory efficiency [12]. Values above 35 accompanied by a low VO_2peak_ can result in poor prognosis in ACHD [22]. In our study, a progressive increase in the VE/VCO_2_ slope occurred in association with VO_2peak_ decrease. This observation underlines the potential importance of studying the mechanisms responsible for this clinical parameter.

Contrary to expectations, this study did not find a significant difference in the EP between baseline and follow-up period. Muller et al. [5] presented EP results which were similar to our study but with EP measured by the load peak (watts). There were no significant differences in EP between baseline and a follow-up period of 2.5 years. Nevertheless, when the peak considered the patient’s body weight, they observed a decreased mean of −0.13 watts.kg between same periods. These findings are in accordance with our study and highlight the importance of controlling peripheral factors such as body weight in the assessment of AC and EP.

One reason for the favorable results of EP in our study could be that our CPET department is extremely engaged in PA and sports participation promotion. After first CPET, all patients receive advice about what kind of physical exercise is safe and useful. This attitude could have influenced the activity lifestyle of participants during follow-up. Unfortunately, exercise participation data has not been provided in the clinical data of all participants. Nerveless, we could include a subgroup of 39 participants with PA assessed by an IPAQ at follow-up. In this study, we cannot conclude that there is a direct relationship between PA and AC. However, our findings are consistent with our previous study [23] and some current studies reporting a positive association between PA and AC, emphasizing that increasing PA practice can improve AC in CHD population [4,24,25,26].

Another explanation is very speculative and it could be related to the management of CPET protocol. Patients were instructed not to hold onto the treadmill safety bar, but in some cases they did, and a possible explanation could be patients’ lack of balance or lack of experience in CPET on a treadmill. In this case, it would be relevant to record information about the position of the hands during the test and, if holding on to the safety bar, consider it in the final assessment of peak VO_2_ consumption.

The limitations of this study are inherent and common to all retrospective studies. We emphasize the lack of PA data in the clinical database. We believe that physical activity data, as well as interventions (events, ablations and interventional catheterization), should be included in the analysis model to determine whether the reduction in AC is due to physical deterioration and/or evolution of heart disease.

Despite this, the strength of our study is that, regardless of the unavailable variables, studying the trend of the gold standard AC parameter (VO_2peak_) can predict the prognosis of the CHD population [22,27,28,29,30]. Nevertheless, these data must be interpreted with caution due to the brief (4.5 years) period of follow-up reported in our study. Moodie D. [31] suggests that proper follow-up needs to find and track CHD patients over 30 to 50 years. In this line, future research with a longer follow-up is needed to obtain consistent results about AC evolution in ACHD population.

## 5. Conclusions

AC in ACHD patients declined during follow-up. This decline was not related to body weight change and the decrease in the EF. Despite this, the HR_peak_ and the EP (duration of exercise time, velocity and percentage of grade) were maintained without a deterioration over time. These results suggest a sign of favorable functional cardiac evolution of the ACHD population. More research is needed, including the level of physical activity of ACHD, in addition to all intervention procedures and events occurring during the follow-up period, to study the contribution of each factor to AC reduction.

## Figures and Tables

**Table 1:** Distribution of participants by Severity class * (n = 127).

Anatomic	Functional	N	%
Moderate (n = 75)	2A	5	0.8
	2B	6	4.7
	2C	63	49.6
	2D	1	0.8
Complex (n = 52)	3A	3	2.4
	3B	14	11.0
	3C	35	27.6

* Severity class by ACC guidelines [2].

**Table 2:** Distribution of cases by diagnostic group (n = 127).

Diagnostic	N	%
Tetralogy of Fallot	25	31.8
Transposition of the great arteries	30	38.1
Pulmonary valve stenosis	15	19.1
Univentricular heart	13	16.5
Aortic stenosis	12	15.2
Atrioventricular canal defect	7	8.9
Ebstein’s anomaly	7	8.9
Ventricular septal defect	4	5.1
Atrial septal defect	3	3.8
Scimitar syndrome	2	2.5
Pulmonary atresia	3	3.8
Miscellaneous *	6	7.6

* Truncus arteriosus (1); Coronary anomaly (1); Tricuspid dysplasia (1); Patent ductus arteriosus (1); Multiple diagnosis (1); Mitral valve cleft (1).

**Table 3:** Baseline characteristics of CHD participants.

	Moderate n = 75			Complex n = 52			Total n = 127		
Variable	Baseline	Follow-Up	Change	Baseline	Follow-Up	Change	Baseline	Follow-Up	Change
Gender (% Female)	61.8%			50.0%			57.5%		
Age (years)	35.7 (11.7)	40.5 (11.6)	4.7 (1.9)	31.1 (9.5)	35.6 (10.0)	4.5 (2.3)	33.8 (11.1)	38.5 (12.2)	4.6 (2.1) ^a^
Height (cm)	167.0 (0.1)	167.0 (0.1)	-	165.0 (0.1)	165.0 (0.1)	-	166.0 (0.1)	166.0 (0.1)	
Weight (kg)	66.1 (13.1)	67.4 (13.2)	1.2 (5.9)	62.8 (12.2)	64.8 (13.3)	2.0 (4.1)	64.8 (12.8)	66.3 (23.3)	1.5 (18.0) ^b^
BMI (kg/m^2^)	23.5 (3.6)	23.94 (3.8)	0.4 (1.8)	22.8 (3.5)	23.4 (3.8)	0.6 (1.5)	23.2 (3.6)	23.7 (3.8)	0.5 (3.7) ^b^
**Clinical Data**									
% Ejection fraction	63.5 (9.2)	59.7 (7.1)	−3.7 (7.8)	50.1 (15.4)	47.6 (12.6)	−3.0 (9.8)	59.6 (12.8)	55.2 (11.1)	−3.5 (8.2) ^a,b^
Beta-bloquer n (%)	8 (6.3%)	16 (12.6%)	8	8 (6.3%)	16 (12.6%)	8	16.0 (12.6%)	32.0 (25.2)	16 ^b^
ACE/Diuretics n (%)	5 (3.9%)	11 (8.7%)	6	5 (3.9%)	8 (6.3%)	3	10.0 (7.9%)	19.0 (15.0)	9 ^b^
**CPET data**									
VE/VCO_2_ slope	26.0 (5.9)	27.2 (4.7)	1.2 (0.6)	28.1 (7.3)	30.2 (8.4)	2.1 (0.7)	26.9 (6.6)	28.5 (6.7)	1.6 (4.9) ^a,b^
% VO_2_ predicted	87.6 (21.4)	85.8 (19.9)	−1.8(16.8)	76.1 (16.4)	70.0 (18.5)	−6.0 (13.3)	82.9 (20.3)	79.3 (20.8)	−3.5 (15.7) ^a,b^
VO_2_ absolute (L/min)	1.8 (0.6)	1.9 (0.7)	0.01 (0.03)	1.7 (0.5)	1.6 (0.5)	−0.1 (0.3)	1.8 (0.6)	1.8 (0.6)	−0.03 (0.36) ^a^
VO_2peak_ (mL/kg/min)	28.3 (8.4)	27.8 (8.4)	−0.5 (5.6)	27.2 (6.5)	24.8 (6.7)	−2.3 (4.7)	27.8 (27.7)	26.6 (7.8)	−1.3 (5.3) ^b^
Peak O_2_ pulse	11.4 (3.8)	11.5 (3.4)	0.1 (2.5)	11.1 (3.7)	10.6 (3.7)	−0.5 (1.9)	11.2 (3.7)	11.1 (3.6)	−0.1(2.8)
HR_peak_ (bpm)	163.0 (19.6)	161.9 (21.2)	−1.1 (16.4)	156.3 (22.7)	154.2 (23.4)	−2.1 (15.9)	160.3 (21.1)	158.8 (22.3)	−1.5(16.0) ^a^
Time exercise (minutes)	13.0 (3.4)	12.6 (2.7))	−0.4 (2.8)	12.6 (2.8)	12.4 (2.4)	−0.2 (1.9)	12.8 (3.2)	12.6 (2.7)	−0.3 (2.5)
Percentage slope (%)	11.4 (1.5)	11.6 (1.2)	0.2 (1.5)	11.6 (1.0)	11.8 (0.7)	0.2 (0.9)	11.5 (1.3)	11.7 (1.0)	0.2 (1.3)
Maximal Velocity (km/h)	6.3 (1.1)	6.2 (0.9)	−0.1 (0.8)	6.2 (0.9)	6.0 (0.9)	−0.2 (0.6)	6.2 (1.0)	6.1 (0.9)	−0.1 (0.7)

Note: BMI, body mass index. Values are presented as mean and standard deviation (SD). ^a^ Comparing between severity CHD groups *p* < 0.05. ^b^ Comparing baseline values with follow-up by a post-hoc comparison, two-way ANOVA *p* < 0.001 except for VO_2peak_ *p* = 0.003.

## Data Availability

Not applicable.
